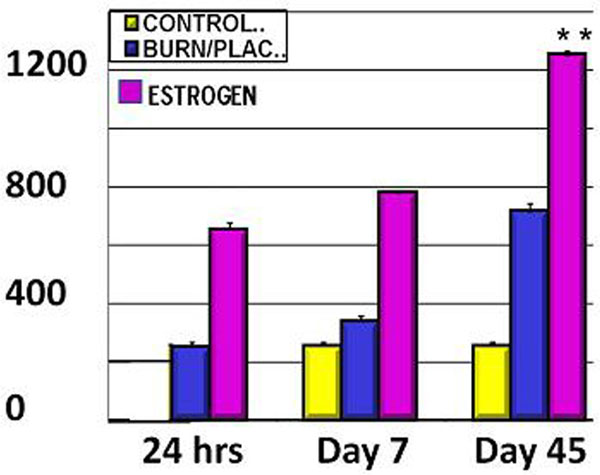# Single-dose estrogen infusion can amplify brain levels of Sonic hedgehog, a signal protein for neuro stem cells and repair following the indirect brain injury resulting after severe torso burns

**DOI:** 10.1186/cc12225

**Published:** 2013-03-19

**Authors:** PE Pepe, JG Wigginton, JW Gatson, J Simpkins, D Maass, K AbdelFattah, AH Idris, V Warren, JP Minei

**Affiliations:** 1 University of Texas Southwestern Medical Center, Dallas, TX, USA; 2 University of North Texas, Fort Worth, TX, USA

## Introduction

Severe burn patients are often noted to have subsequent neurocognitive problems. Experimentally, we have found striking, prolonged elevations of inflammatory markers in the brain (for example, IL-6) even when the injury occurs in a remote anatomic location. This neuroinflammatory response can also be significantly blunted by a single post-burn dose of estrogen. Sonic hedgehog (SHH), an important signaling protein found in the brain, controls and directs differentiation of neural stem cells, influencing brain regeneration and repair by generating new neurons throughout life. As estrogens not only blunt inflammation but also exert an influence on a variety of stem cells, we hypothesized that 17β-estradiol (E2) might affect levels of SHH in the post-burn rat brain.

## Methods

Male rats (*n = *44) were assigned randomly into three groups: controls/no burn (*n = *4); burn/placebo (*n = *20); and burn/E2 (*n = *20). Burned rats received a 40% 3^° ^TBSA dorsal burn, fluid resuscitation and one dose of E2 or placebo (0.5 mg/kg intraperitoneally) 15 minutes post burn. Eight animals from each of the two burn groups (burn/placebo and burn/E2) were sacrificed at 24 hours and at 7 days, respectively (sham group at 7 days only), with four each of the two burn groups sacrificed at 45 days. Brain tissue samples were analyzed by ELISA for SHH.

## Results

Mean levels of SHH levels were significantly elevated within 24 hours as much as 45 days post injury in burned animals receiving the 17β-estradiol (>1,200 pcg/mg) as compared with the placebo-treated burned animals (<700 pg/mg) and controls (<300 pcg/mg). See Figure 1.

## Conclusion

Early, single-dose estrogen administration following severe burn injury significantly elevated levels of SHH in brain tissue. This finding may represent an extremely novel and important pathway for both neuroprotection and neuroregeneration in burn patients.

**Figure 1:**